# Health and Nutrition Analysis in Older Adults in San José de Minas Rural Parish in Quito, Ecuador

**DOI:** 10.1155/2023/1839084

**Published:** 2023-02-13

**Authors:** Roberto Ordoñez-Araque, Carla Caicedo-Jaramillo, Meybol Gessa-Gálvez, José Proaño-Zavala

**Affiliations:** ^1^Facultad de Salud y Bienestar, Escuela de Nutrición y Dietética, Universidad Iberoamericana Del Ecuador (UNIBE), Quito, Ecuador; ^2^Escuela de Gastronomía, Universidad de Las Américas (UDLA), Quito, Ecuador; ^3^ESAI Business School, Universidad Espíritu Santo, Guayaquil, Ecuador

## Abstract

Knowing the health and nutritional status of older adults is crucial to helping them live healthier lives and limiting the need for pharmaceuticals and complicated medical procedures. The objective of this research was to analyze the eating habits (EH), physical activity (PA), and sleep quality (SQ) of older adults in the rural parish of San José de Minas in Quito, Ecuador. Three validated questionnaires were used: the Pittsburgh PSQI for SQ, IPAQ for PA, and frequency of consumption for EH. The results revealed high consumption of refined flours and sugar (70% at least once a day), low intake of whole grains, fish, and olive oil, and considerable consumption of fruits and water. Fifty percent of respondents engage in moderate physical activity and 24% in low physical activity, while 90% of older adults have poor sleep quality. These results indicate a problem in the integral health of the population that does not allow older adults to have a good old age. Health campaigns should be developed to increase physical activity, encourage a better diet, and thus, improve the quality of sleep.

## 1. Introduction

In 2010, the most recent census conducted in Ecuador showed that 6.5% of the population was older than 65 years. The projection (in Ecuador, there is no current population data; a census will be carried out in December 2022) indicated that by 2017, this could increase to 7%, and by 2050, to 17% [1]. These data suggest that close attention should be paid to the health of older adults, who form an increasingly large proportion of society. Unfortunately, the last health census in Ecuador did not rather assess the nutritional status of adults over 60 years of age, but it is reported that 6 out of 10 would be overweight or obese, a figure that resembles the population aged between 19 and 59 years; this may occur mainly due to the poor eating habits of Ecuador's adult population, where the staple foods in the daily diet consist of rice, white bread, refined palm oil, and sugar [2].

Older adults must constantly perform daily physical activities, and these should be focused on the needs of their age. In the area of health, they need to specifically take into account their physical activity and eating habits [3]. One of the main strategies for optimum aging is to practice mild to moderate physical activity. Several studies have shown that exercising at least three times a week can improve an individual's functional capacity and effectively improve performance and autonomy in basic daily activities [4]. Physical activity also contributes to improvements in intellectual capacity, strength, flexibility, and aerobic capacity [5]. In addition, it improves the immune system response, can reduce the risk of falls and cardiovascular diseases, can contribute to a reduction in muscle atrophy, increases the excretion of cholesterol, and consequently reduces the risk of atherosclerosis [4, 6, 7].

Eating habits are a key element in the health of older adults, and knowing and choosing foods for a healthy daily diet is essential because of the different nutritional needs of this segment of the population. In general, older adults require a higher intake of proteins of high biological value, accompanied by the daily recommended intake of vitamins (mainly vitamin C and B complex), minerals (especially calcium, magnesium, iron, and zinc), and fiber. Nutrient deficiency or excess can cause metabolic and psychological alterations [8, 9]. It should be noted that access to good nutritional choices often depends on individual or family resources. In Latin American countries such as Ecuador, this may depend on knowledge of nutrition at the time of purchase as, to a certain extent, vegetable foods may be cheaper than processed foods [8, 10].

Finally, more aspects that can determine the overall health of older adults should be analyzed. Because of its relationship with physical activity, nutrition, and mental health, quality of sleep must be considered [11]. Insomnia is usually prevalent among older adults. This disorder, along with poor sleeping conditions, can lead to lack of concentration and difficulty paying attention, a decrease in capacity and physical activity, complications with existing pathologies, and deteriorating mental health since poor sleep can be related to psychological and social problems [11, 12].

The objective of this study was to determine the integral health of older adults in a rural parish in the city of Quito, Ecuador, by studying their eating habits, physical activity, and current sleep quality. The results will provide evidence of the health of elderly people in a rural population of Ecuador and will allow us to analyze whether better attention is needed from the country's health authorities in this demographic group.

## 2. Materials and Methods

### 2.1. Study Population and Sample

This study was carried out in the parish of San José de Minas, located 80 kilometers from the city of Quito, in the province of Pichincha, Ecuador. According to the most recent population and housing census, conducted in 2010 by the National Institute of Statistics and Census (INEC) of Ecuador, this rural parish has 7243 inhabitants, with a projected population of 8859 inhabitants in 2019. At the 2010 census, the parish had 952 adults aged 65 years and older [1]. San José de Minas is situated at 78°, 24′, 30″ west, and 0° 11′ north. It has an altitude range between 1200 and 3580 meters above sea level, and its territory is made up of 26 neighborhoods. Through the decentralized autonomous government (GAD) of the parish, the elderly population was invited to participate in this research. The GAD granted the researchers permission to work with the Centro neighborhood which is home to 70 elderly people. The sample corresponded to people aged 65years or older who wished to participate. A total of 60 people participated in the research. They were asked to provide sociodemographic information (sex, age, and level of education), and they participated in three validated questionnaire surveys that sought to obtain information about physical activity, quality of sleep, and food consumption habits. The participants were informed of the objectives of the study and signed a consent form. The members of the research team of this study assisted the participants to complete the surveys and helped resolve any questions concerning different sections of the questionnaires. Research manuscripts reporting large datasets that are deposited in a publicly available database should specify where the data have been deposited and provide the relevant accession numbers. If the accession numbers have not yet been obtained at the time of submission, please state that they will be provided during review. They must be provided prior to publication.

### 2.2. Instruments

#### 2.2.1. Eating Habits

To analyze dietary habits, the Food Group Consumption Frequency Questionnaire, developed and validated by Goni et al. in 2016, was used [13]. This questionnaire is based on the exchange system, in which portions are defined according to their energy content and number of macronutrients and includes 19 food groups: whole dairy, semiskimmed and skimmed dairy, eggs, lean meats, fatty meats, white fish, oily fish, vegetables, fruits, nuts, legumes, olive oil, other fats, refined cereals, whole grains, industrial pastries, free sugars, alcohol, and water. In this instrument, respondents indicated the frequency of consumption as follows: never or almost never, times per month, times per week, or times per day.

#### 2.2.2. Physical Activity

The short version of the International Physical Activity Questionnaire (IPAQ) was used to analyze physical activity. The reliability and validity of this instrument have been proven in different national and regional studies worldwide, and the World Health Organization (WHO) recommends its use in the measurement of physical activity in epidemiological surveillance at the population level [14]. This questionnaire consists of questions about physical habits; the answers are tabulated with the number of minutes, hours, or days of physical activity. The unit of measurement used for the result is Mets (metabolic equivalent of task), and physical activity is classified as high or vigorous (AFA), moderate (AFM), and low or walking (AFB). Questions 1 and 2 are used to calculate AFA by multiplying 8 MET x minutes x days per week. Questions 3 and 4 are used to calculate AFM by multiplying 4 MET x minutes x days per week. Questions 5 and 6 are used to calculate AFB by multiplying 3.3 MET × minutes × days per week. Question 7 is a reference to the time the respondent spends sitting and is not used in the calculation. A score of 1500 to 3000 MET or more signifies AFA, 600 to 1500 MET signifies AFM, and less than 600 MET signifies AFB [14, 15].

#### 2.2.3. Sleep Quality

The Pittsburgh Sleep Quality Index (PSQI) questionnaire was used to measure sleep quality. This questionnaire was developed in 1988 in the Department of Psychiatry of the University of Pittsburgh and, due to its effectiveness, has been used in several investigations with different populations. This instrument evaluates quantitative and qualitative aspects of sleep quality over the preceding month [16]. The questionnaire consists of 24 questions, 19 of which are self-administered and 5 of which are answered by an individual who lives with the respondent. Eighteen of the self-administered questions were used for the tabulation, with components that evaluate time of going to bed and time taken to fall asleep, time of waking, approximate hours of sleep, causes that provoke problems with time taken to fall asleep (e.g., not being able to fall asleep immediately upon going to bed, getting up suddenly for no reason or to go to the bathroom, not breathing well, coughing or snoring loudly, excessive cold or heat, nightmares, and suffering from pain), assessment of sleep quality, consumption of sleep medications, feeling drowsy when driving or performing daily activities, and having the desire to perform different activities. The questions were evaluated according to established indicators, with scores ranging from 0 (no problems) to 3 (serious problems). After tabulation, 7 scores were obtained from all the questions for a possible total sum of 21 points; a score from 0 to 5 indicates good quality of sleep, while a score of more than 5 indicates sleep problems [16, 17].

#### 2.2.4. Statistical Analysis

Data from the questionnaires were analyzed, with descriptive statistics used to determine the existence of statistically significant differences in the qualitative variables (quality of sleep and physical activity), using repeated measures analysis of variance (ANOVA). The quantitative variables of sleep quality and physical activity were expressed as percentages and absolute frequencies. For the results of frequency related to consumption, foods were analyzed according to the responses obtained to compare percentages and to analyze eating habits, which were grouped according to the highest response percentages in the questionnaire. Finally, to determine the correlation between sleep quality and physical activity, Pearson's test was used to check for the existence of a relationship between the variables. SPSS, version 15.0 for Windows (Statistical Package for the Social Sciences; Chicago, Illinois), was used to conduct statistical analyses.

## 3. Results

### 3.1. Sociodemographic Variables

The population sample consisted of 60 individuals, 50% female and 50% male (percentage that can be related to the projected total population of older adults by 2020 in Ecuador: 54% female and 46% male), ranging in age from 65 to 89 years. The majority of respondents had completed primary education or school (78%).  Table 1 shows the detailed characteristics of the study population.

### 3.2. Eating Habits

**Figure 1:**
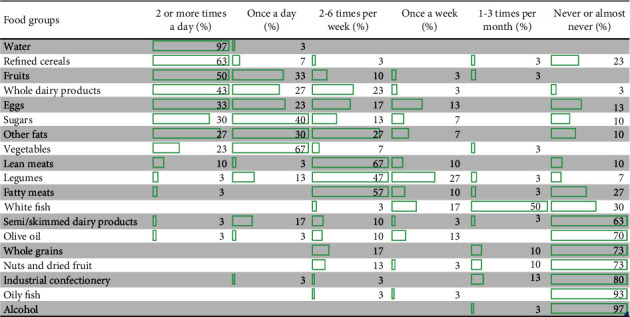
Frequency of food consumption in older adults of the San José de Minas parish.

Figure 1 shows the frequency of food consumption of the elderly population sample of the parish. In general, we can observe a high consumption of water (twice per day) in the entire population (97%). Among the population, 63% have high refined flour consumption, while whole grains are not considered an important component of the diet (73% never or almost never). Fruit is consumed twice a day or more by 50% of the population, while 67% consume vegetables once a day. Whole milk products are consumed twice or more per day by 43% of respondents, while skimmed milk products are never or almost never consumed by 63%. Eggs are consumed twice a day or more by 33% of respondents and once a day by 23%. There is a high incidence of sugar consumption, with 40% consuming sugar once a day and 30% twice a day or more. Consumption of confectionery is low, with 80% of respondents stating they never or almost never buy it. Olive oil is never consumed by 70% of the population, while other types of fats are consumed once a day by 30% and twice a day or more by 27%. Lean meats and fats are consumed between 2 and 6 times per week by 67% and 57% of respondents, respectively. Legumes are consumed between 2 and 6 times per week by 47% of the population. In the parish, 50% of respondents report consuming white fish between 1 and 3 times per month, while 93% never or almost never consume oily fish. Finally, 73% of respondents never or almost never eat nuts, while 97% never or almost never consume alcohol.

The food groups are situated according to the main results obtained.

### 3.3. Physical Activity

Table 2 shows the results of the physical activity survey. 24% of older adults engage in low physical activity (272.5 METs), 50% engage in moderate physical activity (on average 1086.5 METs), and 27% engage in high physical activity (3684.75 METs).

In the individual results, it can be seen that women (57%-1365.9 METs) do more moderate physical activity than men (43%-1131.58 METs). The highest METs difference between genders is found in high physical activity; 69% of men have an average of metabolic equivalent minutes per week of 4160.4, compared to 31% of women with an average metabolic equivalent minutes per week of 2892. Finally, 57% of women engage in low physical activity (359.33 METs) compared to 43% of men (305.5 METs).

### 3.4. Sleep Quality

Table 2 shows the results obtained for quality of sleep. Most of the population sample (90%) experiences sleeping problems. This result is reflected in both men and women; only 4 female respondents and 2 male respondents did not report sleep problems.

### 3.5. Physical Activity and Sleep Quality

**Figure 2:**
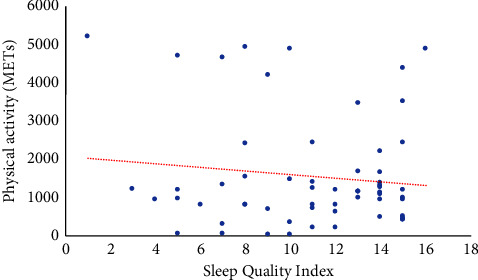
Relationship between physical activity and sleep quality.

A correlation graph was created to analyze the relationship between the variables of physical activity and sleep quality. The correlation coefficient of −0.003 for Spearman's test was obtained (the coefficient of determination (*R*^2^) obtained was 0.0148). These values indicate a negative correlation, i.e., the variables have no relationship. This is reflected in the fact that 90% of respondents have poor quality of sleep independent of the physical activity they perform. It should be noted that there was a slight correlation for Pearson's test, indicating that, to some extent, individuals with poor quality sleep also engage in low physical activity (Figure 2).

## 4. Discussion

Elderly people represent one of the most vulnerable age groups in society. According to the latest WHO reports, by 2030, the population over 60 years of age will increase by 34% due to higher life expectancy. However, this does not mean that people are healthy in their later years; on the contrary, there is evidence of the globalization of aging, aggravated by health systems that are unprepared for a larger population of older adults [18]. For this reason, the study and analysis of the health status of the elderly are essential, so that preventive measures can be integrated into old age. In Ecuador, authorities need to become more aware of this important stage in life and begin to see the reality of older adults in different populations given that they were not included in the last nutrition survey conducted in the country [2].

In general, demographic characteristics influence the aging of the population. For instance, low levels of educational attainment in older adults, as observed in the population sample studied (78% completed school only), can expose them to significant social and economic adversity, which can affect self-care attributes such as eating habits, sleep, and disease as demonstrated by other research [18, 19]. This can be compared with the study by Salazar-Barajas et al. in Matamoros in Tamaulipas, Mexico, where older adults who lacked advanced schooling reported low physical activity and poor eating habits, leading to the prevalence of chronic diseases and, in particular, hypertension [20].

It is important to take the eating habits of older adults into account, as this demographic is directly affected by their diet throughout the life cycle, especially during adulthood. Dietary habits, together with normal physical and cognitive deterioration, may influence the quality of life in older adulthood. A study by Mendoza and Márquez in Chimbote, Peru, found that most of the older adult population (60.8%) had an unhealthy lifestyle, and 58% had some form of malnutrition [21]. In this sense, food transition plays an important role as most age groups are currently exposed to energy-dense foods that are high in sugar and sodium, low in nutritional quality, and easy to prepare. This can become a risk factor for older adults given that in this vulnerable group high nutritional quality foods should be prioritized due to age-related changes [22, 23].

The changes to which older adults are exposed during the aging process should be taken into account. By nature, organs deteriorate and their functioning changes, starting with the oral cavity, which can alter the consumption and swallowing of food. Older adults, therefore, may prefer foods that are easy to swallow or that present pronounced sweet or salty flavors which may have limited nutritional value [22, 24]. The results from this research show that 70% of the older population sample of San José de Minas consume sugars and refined flours daily, compared to 73% of those who never consume whole grains. In this regard, it should be noted that the high intake of added sugars increases the risk of mortality and the appearance of a variety of diseases [25], while the consumption of whole grains is related to numerous health benefits and improved aging. Indeed, current recommendations indicate that the consumption of whole grains should be promoted over that of refined flour and sugar, without increasing energy intake [26]. In addition to high consumption of refined cereals and sugars and low consumption of whole grains, the older population of San José de Minas reported low consumption of olive oil, nuts, legumes, and animal proteins. Their diet, therefore, can be considered inadequate and the basis for the appearance of a variety of pathologies in the elderly, including problems in sleep quality [27]. In general, it can be determined that the current diet of older adults in the parish is not adequate. Dietary recommendations for older adults should emphasize an increase in the consumption of whole fruits, vegetables, greens and beans, whole grains, dairy, protein foods, seafood and plant proteins, and fatty acid, and a decrease in the consumption of refined grains, sodium, added sugar, and saturated fats [23].

In terms of exercise, 74% of the population sample reported moderate to low physical activity, while only 27% reported high physical activity. In San José de Minas, older adults mainly engage in walking. The lack of physical activity in the elderly is related to chronic diseases such as diabetes mellitus, hypertension, sarcopenia, heart disease, and cancer, while a lack of physical activity combined with a poor diet leads to the prevalence of weight gain and obesity. For this reason, it is essential to increase regular physical activity and aerobic exercise, which are associated with a decrease in mortality from all causes, improvements in mental health and cognitive function, the prevention of dementia, improvements in sleep quality, prevention of falls, and a lower prevalence of diseases in general [28].

In spite of not evaluating the prevalence of certain diseases in the study population, important data can be extrapolated from many articles. Lack of sleep or poor sleep quality, unhealthy diet, and sedentarism are confirmed risk factors for a variety of noncommunicable chronic diseases such as diabetes, cardiovascular disease, and hypertension [29–32] besides common elderly diseases such as sarcopenia or dementia [33–36]. Some studies and metanalyses made in elderly people [37–39] conclude that both fat mass and physical activity are considered risk factors for muscle mass quality and sarcopenia. For example, the study performed by Lee et al. [40] establishes that body fat and lack of exercise modulates the association between sarcopenia and osteoporosis as these are frequent illnesses among older people. That same study determines high body fat as an independent risk factor for osteoporotic fractures, which overall indicates that lifestyle habits do in fact influence elderly health. One of the major causes of sarcopenia is the depletion of muscle mass which is caused by a lack of physical activity and a careless diet. In this matter, a healthy diet is important in these age groups, as stated in the umbrella review by Gielen et al. [41], and protein supplementation, especially leucine, hand in hand with strength and resistance training can have a significant effect on muscle mass and strength.

It is safe to say that as age advances, the signs and symptoms of disease also increase that is why a multicomponent approach is needed to evaluate the elderly, as it is a fact that a combination of modifiable lifestyle factors such as diet, sleep quality, and physical activity are definitely accurate predictors for different diseases, for example, some kinds of dementia [33, 34].

It is recommended that older adults perform 75 minutes of high and 150 minutes of moderate aerobic activity per week and engage in muscle strengthening activities at least twice per week (it is important that possible existing cardiac problems are analyzed for the recommendation of personalized exercise regimes) [42]. Both men and women in the parish report engaging in moderate or low physical activity. This is because their activities are routine, and most older adults do not perform new activities. It is important that location is taken into account since rural regions may have limited access to the resources, services, equipment, and transportation required to implement physical activity programs. Authorities should include in their policies group exercise programs that involve the entire community and prioritize older adults. Such programs could be carried out in community halls and preferably be free of charge. It is also important to form groups that are welcoming to all genders, in order to encourage the participation of men, given that they participate in exercise less than women [43].

Although several factors, such as depression, clinical diseases, polypharmacy, family, environmental, and social problems, impact the sleep of older adults, lifestyle habits also play a very important role [44]. In this context, the older adults of the parish do not perform physical activity according to health recommendations. It has been shown that exercise programs with a frequency of 3 times per week can significantly improve the sleep of older adults, thus avoiding fatigue and improving the general functioning of the body, mental health, and even the desire to engage in physical activity [45]. The dietary habits observed in the study population sample can have an impact on the sleep quality of older adults (90%). These results can be compared with Wilson et al.'s findings on the influence on sleep of the quality of fats and carbohydrates consumed, in which diets high in complex carbohydrates, healthy fats, protein, and anti-inflammatory nutrients are associated with better sleep quality [46]. Likewise, several studies [47, 48] have found that foods that promote sleep, such as avocado, eggs, meats, and oily fish, are high in tryptophan (an amino acid precursor of hormones involved in sleep). These are precisely the foods that the study population consumes the least. These same studies also indicate that an unhealthy diet is associated with shorter sleep time and irregular sleep patterns. These same habits, which can be considered inadequate, translate into a diet with a high glycemic index, which represents a high-risk factor for insomnia [49, 50]; that is, it affects the quantity and quality of sleep, especially in older adults who already experience more delicate sleep. It should also be noted that dietary habits and physical activity may have changed during the COVID-19 health emergency, leading to altered sleep patterns. In Ecuador, specifically, it has been found that during the pandemic, both older men and women experienced deficiencies in sleep quality [51].

Due to the aging of the world population, it is very important that the health of the elderly is taken into account [18]. Physical activity, healthy diet, and adequate sleep, together with good mental health and active participation in society, have been found to delay aging [52] and ensure a good quality of life for this age group.

It is important to mention that although the results obtained show how the older adults of the parish need to improve their health, the data may have several limitations: the sample used may not necessarily represent all the older adults of the parish; it would be important to determine anthropometric indicators; and it is also necessary to conduct a health outcome study to determine whether the results show correlation or causality.

## 5. Conclusions

With the data obtained, the GAD should promote physical activity and nutrition programs for older adults in the parish of San José de Minas. Programs should be focused on groups of adults or, if possible, include the families of older adults, to achieve permanence in the programs and to encourage the creation of habits that will continue outside of the programs.

The study found that older adults do not have good eating habits and, for the most part, they choose foods that do not provide nutritional value. The study also found that there is a need for increased physical activity. Improving these two factors can increase the sleep quality of almost all older adults who, at present, have problems sleeping.

Although there is no direct correlation between physical activity and sleep quality, it can be noted that people who engage in limited exercise are more prone to sleep problems; for this reason, older adults should be encouraged to engage in physical activity.

## Figures and Tables

**Table 1:** Sociodemographic variables of San José de Minas parish.

	Male	Female	Total
Years			
65–69 years	3 (5%)	6 (10%)	9 (15%)
70–74 years	5 (8%)	4 (7%)	9 (15%)
75–79 years	9 (15%)	11 (18%)	20 (33%)
80–84 years	9 (15%)	6 (10%)	15 (25%)
85–89 years	4 (7%)	3 (5%)	7 (12%)
Total	30 (50%)	30 (50%)	60 (100%)
Education			
Elementary school	22 (37%)	25 (42%)	47 (78%)
Incomplete high school	5 (8%)	4 (7%)	9 (15%)
High school	2 (3%)	1 (2%)	3 (5%)
University	1 (2%)	0 (0%)	1 (2%)
Total	30 (50%)	30 (50%)	60 (100%)

**Table 2:** Sleep quality and physical activity in older adults of the San José de Minas parish.

	65–69 years	70–74 years	75–79 years	80–84 years	85–89 years	Female	Male	Total
Sleep quality *n* (%)								
Good sleepers	2 (3.33%)	2 (3.33%)	2 (3.33%)	0 (0%)	0 (0%)	4 (6.67%)	2 (3.33%)	6 (10%)
Poor sleepers	7 (11.67%)	7 (11.67%)	18 (30%)	15 (25%)	7 (11.67%)	26 (43.33%)	28 (46.67%)	54 (90%)
Total	15%	15%	33%	25%	12%	30 (50%)	30 (50%)	60 (100%)
Physical activity *n* (%)								
High	3 (18.75%)	3 (18.75%)	6 (37.50%)	3 (18.75%)	1 (6.25%)	5 (31.25%)	11 (68.75%)	16 (27%)
Median MET-min/wk (IQR)	3519	5190	3583	4857	1638	2892 (870–7200)	4160,4 (1800–12560)	3684.75 (1220–8420)
Moderate	4 (13.33%)	5 (16.67%)	12 (40%)	5 (16.67%)	4 (13.33%)	17 (56.67%)	13 (43.33%)	30 (50%)
Median MET-min/wk (IQR)	936.75	1089	1188.58	988.5	1024.5	1365.9 (1010–3500)	1131.58 (1920–6700)	1086.5 (1150–4200)
Low	2 (14.29%)	1 (7.14%)	2 (14.29%)	7 (50%)	2 (14.29%)	8 (57.14%)	6 (42.86%)	14 (24%)
Median MET-min/wk (IQR)	240.5	40.5	150.2	361.5	396	359.33 (210–500)	305.5 (250–670)	272.5 (350–605)
Total	15%	15%	33%	25%	12%	50%	50%	60 (100%)

Sleep quality results of individuals with poor sleep quality and moderate physical activity prevailed (*p* < 0.01) in both male and female respondents. Two decimal places were used for percentages. MET-min/wk: metabolic equivalent minutes per week. IQR: interquartile range. The IQR is found in the global total and the total for males and females.

## Data Availability

The data supporting the findings of the current study are available from the corresponding author upon request.

## References

[1] INEC (2010). Resultados del censo 2010 Ecuador. *Inst Ecuatoriano Estadística y Censos*.

[2] FAO (2013). Encuesta nacional de salud y nutrición del Ecuador (ENSANUT) FAO. http://www.fao.org/family-farming/detail/es/c/317359/.

[3] Štefan L., Petrinović L., Sporiš G., Vrgoč G. (2018). Frequency of dietary intake and physical activity in older adults: a cross-sectional study. *Nutrients*.

[4] Son J., Nimrod G., West S., Janke M., Liechty T., Naar J. (2021). Promoting older adults’ physical activity and social well-being during covid-19. *Leisure Sciences*.

[5] Gammack J. (2017). Physical activity in older persons. *Missouri Medicine*.

[6] Rivera-Torres S., Fahey T. D., Rivera M. A. (2019). Adherence to exercise programs in older adults: informative report. *Gerontology and Geriatric Medicine*.

[7] Kelley G. A., Kelley K. S., Tran Z. V. (2005). Exercise, lipids, and lipoproteins in older adults: a meta-analysis. *Preventive Cardiology*.

[8] Zaragoza-Martí A., Ruiz-Robledillo N., Sánchez-Sansegundo M., Albaladejo-Blázquez N., Hurtado-Sánchez J., Ferrer-Cascales R. (2020). Eating habits in older adults: compliance with the recommended daily intakes and its relationship with sociodemographic characteristics, clinical conditions, and lifestyles. *Nutrients*.

[9] Kaur D., Rasane P., Singh J. (2019). Nutritional interventions for elderly and considerations for the development of geriatric foods. *Current Aging Science*.

[10] Romero Viamonte K., Sánchez Martínez B., Vega Falcón V., Salvent Tames A. (2020). Estado nutricional en adultos de población rural en un cantón de la sierra ecuatoriana. *Revista Ciencias de la Salud*.

[11] Thichumpa W., Howteerakul N., Suwannapong N., Tantrakul V. (2018). Sleep quality and associated factors among the elderly living in rural Chiang Rai, northern Thailand. *Epidemiol Health*.

[12] Sun X. H., Ma T., Yao S. (2020). Associations of sleep quality and sleep duration with frailty and pre-frailty in an elderly population Rugao longevity and ageing study. *BMC Geriatrics*.

[13] Goni L., Aray Miranda M., Martínez J. A., Cuervo M. (2016). Validación de un cuestionario de frecuencia de consumo de grupos de alimentos basado en un sistema de intercambios. *Nutricion Hospitalaria*.

[14] Ordoñez-Araque R., Caicedo-Jaramillo C., García-Ulloa M., Dueñas-Ricaurte J. (2021). Eating habits and physical activity before and during the health emergency due to COVID-19 in Quito – Ecuador. *Human Nutrition & Metabolism*.

[15] Oyeyemi A. L., Moss S. J., Monyeki M. A., Kruger H. S. (2016). Measurement of physical activity in urban and rural South African adults: a comparison of two self-report methods. *BMC Public Health*.

[16] Carralero P., Hoyos F. R., Deblas Á, López M. (2013). Calidad del sueño según el Pittsburgh Sleep Quality Index en una muestra de pacientes recibiendo cuidados paliativos. *Medicina Paliativa*.

[17] Faulkner S., Sidey-Gibbons C. (2019). Use of the Pittsburgh Sleep quality index in people with schizophrenia spectrum disorders: a mixed methods study. *Frontiers in Psychiatry*.

[18] Oms (2018). *Envejecimiento Y Salud*.

[19] Leitón Espinoza Z. E., Villanueva Benites M. E., Fajardo Ramos E., Leitón Espinoza Z. E., Villanueva Benites M. E., Fajardo Ramos E. (2019). Relationship between demographic variables and self-caring practices of the elderly adult with diabetes mellitus. *Salud Uninorte*.

[20] Salazar-Barajas M., Salazar-González B., Ávila-Alpirez H., Guerra Ordóñez J., Ruiz Cerino J., Durán-Badillo T. (2020). Hábitos alimentarios y actividad física en adultos mayores con enfermedad crónica. *Cienc y enfermería*.

[21] Torrejón Mendoza C., Reyna Márquez E. (2012). Estilo de vida y estado nutricional del adulto mayor. *In Crescendo*.

[22] Troncoso Pantoja C. (2017). Alimentación del adulto mayor según lugar de residencia Claudia. *Horizonte Médico (Lima)*.

[23] Long T., Zhang K., Chen Y., Wu C. (2022). Trends in diet quality among older US adults from 2001 to 2018. *JAMA Network Open*.

[24] Thompson J. L., Manore M. M., Vaughan L. (2017). *Nutrición, 5ta Edició*.

[25] Barrington W. E., White E. (2016). Mortality outcomes associated with intake of fast-food items and sugar-sweetened drinks among older adults in the Vitamins and Lifestyle (VITAL) study. *Public Health Nutrition*.

[26] Foscolou A., D’Cunha N. M., Naumovski N. (2019). The association between whole grain products consumption and successful aging: a combined analysis of medis and attica epidemiological studies. *Nutrients*.

[27] Gupta C. C., Irwin C., Vincent G. E., Khalesi S. (2021). The relationship between diet and sleep in older adults: a narrative review. *Current Nutrition Reports*.

[28] Izquierdo M., Duque G., Morley J. E. (2021). Physical activity guidelines for older people: knowledge gaps and future directions. *The Lancet Healthy Longevity*.

[29] Fuchs F. D., Whelton P. K. (2019). High blood pressure and cardiovascular disease. *Hypertensionaha*.

[30] Georgieff M. K. (2020). Iron deficiency in pregnancy. *American Journal of Obstetrics and Gynecology*.

[31] Pinna G., Pascale C., La Regina M., Orlandini F. (2012). Hypertension in the elderly. *Italian Journal of Medicine*.

[32] Glovaci D., Fan W., Wong N. D. (2019). Epidemiology of diabetes mellitus and cardiovascular disease. *Current Cardiology Reports*.

[33] Zhao C., Noble J. M., Marder K., Hartman J. S., Gu Y., Scarmeas N. (2018). Dietary patterns, physical activity, sleep, and risk for dementia and cognitive decline. *Curr Nutr Rep*.

[34] Dominguez L. J., Veronese N., Vernuccio L. (2021). Nutrition, physical activity, and other lifestyle factors in the prevention of cognitive decline and dementia. *Nutrients*.

[35] Cruz-Jentoft A. J., Bahat G., Bauer J. (2019). Sarcopenia: revised European consensus on definition and diagnosis. *Age and Ageing*.

[36] Dodds R. M., Roberts H. C., Cooper C., Sayer A. A. (2015). The epidemiology of sarcopenia. *Journal of Clinical Densitometry*.

[37] Bao W., Sun Y., Zhang T. (2020). Exercise programs for muscle mass, muscle strength and physical performance in older adults with sarcopenia: a systematic review and meta-analysis. *Aging and disease*.

[38] Ko J. B., Kim K. B., Shin Y. S. (2021). Predicting sarcopenia of female elderly from physical activity performance measurement using machine learning classifiers. *Clinical Interventions in Aging*.

[39] Petermann-Rocha F., Balntzi V., Gray S. R. (2022). Global prevalence of sarcopenia and severe sarcopenia: a systematic review and meta-analysis. *J Cachexia Sarcopenia Muscle*.

[40] Lee I., Cho J., Jin Y., Ha C., Kim T., Kang H. (2016). Body fat and physical activity modulate the association between sarcopenia and osteoporosis in elderly Korean women. *Journal of Sports Science and Medicine*.

[41] Gielen E., Beckwée D., Delaere A. (2021). Nutritional interventions to improve muscle mass, muscle strength, and physical performance in older people: an umbrella review of systematic reviews and meta-analyses. *Nutrition Reviews*.

[42] CDC (2009). How much physical activity do older adults need?. https://www.cdc.gov/physicalactivity/basics/older_adults/index.htm#:%7E:text=Adults%20aged%2065%20and%20older,hiking%2C%20jogging%2C%20or%20running.

[43] Fien S., Linton C., Mitchell J. S. (2022). Characteristics of community-based exercise programs for community-dwelling older adults in rural/regional areas: a scoping review. *Aging Clinical and Experimental Research*.

[44] Miner B., Kryger M. H. (2017). Sleep in the aging population. *Sleep Medicine Clinics*.

[45] Vanderlinden J., Boen F., van Uffelen J. G. Z. (2020). Effects of physical activity programs on sleep outcomes in older adults: a systematic review. *International Journal of Behavioral Nutrition and Physical Activity*.

[46] Wilson K., St-Onge M.-P., Tasali E. (2022). Diet composition and objectively assessed sleep quality: a narrative review. *Journal of the Academy of Nutrition and Dietetics*.

[47] Canet Sanz T., Jurado Luque M. (2016). Sueño y alimentación. *Sueño Saludab. evidencias y guías actuación*.

[48] Sanlier N., Sabuncular G. (2020). Relationship between nutrition and sleep quality, focusing on the melatonin biosynthesis. *Sleep and Biological Rhythms*.

[49] Gangwisch J. E., Hale L., St-Onge M. P. (2020). High glycemic index and glycemic load diets as risk factors for insomnia: analyses from the Women’s Health Initiative. *The American Journal of Clinical Nutrition*.

[50] Zhao M., Tuo H., Wang S., Zhao L. (2020). The effects of dietary nutrition on sleep and sleep disorders. *Mediators of Inflammation*.

[51] Ramos-Padilla P., Villavicencio-Barriga V. D., Cárdenas-Quintana H., Abril-Merizalde L., Solís-Manzano A., Carpio-Arias T. V. (2021). Eating habits and sleep quality during the COVID-19 pandemic in adult population of Ecuador. *International Journal of Environmental Research and Public Health*.

[52] Aldas-vargas C. A., Chara-Plua N. J., Guerrero-Pluas P. J., Flores-Peña R. (2021). Actividad física en el adulto mayor. *Revista Científica*.

